# Coapplication of Effective Microorganisms and Nanomagnesium Boosts the Agronomic, Physio-Biochemical, Osmolytes, and Antioxidants Defenses Against Salt Stress in *Ipomoea batatas*

**DOI:** 10.3389/fpls.2022.883274

**Published:** 2022-07-13

**Authors:** Taia A. Abd El-Mageed, Mohammed A. H. Gyushi, Khaulood A. Hemida, Mohamed T. El-Saadony, Shimaa A. Abd El-Mageed, Hanan Abdalla, Synan F. AbuQamar, Khaled A. El-Tarabily, Abdelsattar Abdelkhalik

**Affiliations:** ^1^Department of Soil and Water, Faculty of Agriculture, Fayoum University, Fayoum, Egypt; ^2^Department of Horticulture, Faculty of Agriculture, Fayoum University, Fayoum, Egypt; ^3^Department of Botany, Faculty of Science, Fayoum University, Fayoum, Egypt; ^4^Department of Agricultural Microbiology, Faculty of Agriculture, Zagazig University, Zagazig, Egypt; ^5^Department of Agronomy, Faculty of Agriculture, Fayoum University, Fayoum, Egypt; ^6^Department of Botany and Microbiology, Faculty of Science, Zagazig University, Zagazig, Egypt; ^7^Department of Biology, College of Science, United Arab Emirates University, Al-Ain, United Arab Emirates; ^8^Khalifa Center for Genetic Engineering and Biotechnology, United Arab Emirates University, Al-Ain, United Arab Emirates; ^9^Harry Butler Institute, Murdoch University, Murdoch, WA, Australia

**Keywords:** abiotic stress, antioxidant, osmoprotectants, salinity, sweet potato, tuber yield

## Abstract

The application of bio- and nanofertilizers are undoubtedly opening new sustainable approaches toward enhancing abiotic stress tolerance in crops. In this study, we evaluated the application of effective microorganisms (EMs) of five groups belonging to photosynthetic bacteria, lactic acid bacteria, yeast, actinobacteria, and fermenting fungi combined with magnesium oxide (MgO) nanoparticles (MgO-NP) on the growth and productivity of sweet potato plants grown in salt-affected soils. In two field experiments carried out in 2020 and 2021, we tested the impacts of EMs using two treatments (with vs. without EMs as soil drench) coupled with three foliar applications of MgO-NP (0, 50, and 100 μg ml^–1^ of MgO, representing MgO-NP_0_, MgO-NP_50_, and MgO-NP_100_, respectively). In our efforts to investigate the EMs:MgO-NP effects, the performance (growth and yield), nutrient acquisition, and physio-biochemical attributes of sweet potatoes grown in salt-affected soil (7.56 dS m^–1^) were assessed. Our results revealed that salinity stress significantly reduced the growth parameters, yield traits, photosynthetic pigment content (chlorophylls *a* and *b*, and carotenoids), cell membrane stability, relative water content, and nutrient acquisition of sweet potatoes. However, the EMs^+^ and/or MgO-NP-treated plants showed high tolerance to salt stress, specifically with a relatively superior increase when any of the biostimulants were combined. The application of EMs and/or MgO-NP improved osmotic stress tolerance by increasing the relative water content and membrane integrity. These positive responses owed to increase the osmolytes level (proline, free amino acids, and soluble sugars) and antioxidative compounds (non-enzymatic concentration, enzymatic activities, phenolic acid, and carotenoids). We also noticed that soil salinity significantly increased the Na^+^ content, whereas EMS^+^ and/or MgO-NP-treated plants exhibited lower Na^+^ concentration and increased K^+^ concentration and K^+^/Na^+^ ratio. These improvements contributed to increasing the photosynthetic pigments, growth, and yield under salinity stress. The integrative application of EMs and MgO-NP showed higher efficacy bypassing all single treatments. Our findings indicated the potential of coapplying EMs and MgO-NP for future use in attenuating salt-induced damage beneficially promoting crop performance.

## Introduction

Salinity is a severe environmental factor having adverse effects on the growth and productivity of many crops (Kamran et al., 2020; Semida et al., 2021a; Shaaban et al., 2022). Globally, salt-affected soil was recorded as approximately 1,125 million hectares (Shahid et al., 2018; Hossain, 2019). This salinity stress controls several physiological, biochemical, and molecular processes in plants (Munns and Tester, 2008; Gupta and Huang, 2014). High accumulation of soluble salts in the soil generates osmotic stress as a rapid response of the plant, consequently decreasing the absorption capacity of the root system. This stress response also induces alteration in some physiological responses, including membrane interruption and physiological drought, and decrease in stomatal aperture (Munns and Tester, 2008; Hanin et al., 2016). Subsequently, the accumulation of high potential toxic ions, especially Na^+^ and Cl^–^ in plant tissues, has several negative effects in cell metabolism and causes disruption of photosynthesis and respiration, and inhibits the antioxidant machinery (Semida et al., 2015; Abd El-Mageed et al., 2020).

Besides the increased Na^+^ accumulation, the reduction in K^+^ and Ca^+2^ uptakes produced by cytosolic K^+^ and Ca^2+^ efflux impeded cell function, caused cell membrane instability, and hampered enzyme activities (Ahanger et al., 2017; Kamran et al., 2020). Additionally, salinity induces secondary stresses as oxidative stress that produces toxic reactive oxygen species (ROS; O^2^, OH^–^, and H_2_O_2_) (Sarker and Oba, 2020). These ROS damages cellular organelles (membrane, DNA, protein), disturbing several processes such as photosynthesis, transpiration, and stomatal conductance, as well as lowering photosynthetic pigment concentration (Acosta-Motos et al., 2017; Muhammad et al., 2021). Plants react and respond to salinity stress through various pathways such as ion homeostasis and compartmentalization, ions transport, osmotic adaptation, stimulation of antioxidant machinery, and osmolyte biosynthesis (Sarker and Oba, 2020; Semida et al., 2020). These reactions stimulates ROS detoxification, the stabilization of the membrane, mineral uptake, ion distribution, and the ultrastructure of organelles, which increases plant adaptability under saline conditions.

Sweet potato (*Ipomoea batatas* L.) (Lam.) is an herbaceous dicotyledonous plant belonging to the family Convolvulaceae (Byju and George, 2005) with the tuberous root as its most valuable part (Ekanayake and Collins, 2004). Sweet potato tubers are high in carbohydrates, particularly the orange-fleshed tubers being a good source of beta-carotene and vitamin A precursor (Dasgupta et al., 2008). It is ranked seventh among food crops globally; however, the productivity of sweet potato is adversely affected under soil salinity (Dasgupta et al., 2008; Meng et al., 2020).

Developing efficient, ecofriendly, and low-cost pathways for salinity stress management is a major challenge. The potential use of materials such as effective microorganisms (EMs) and nanofertilizer, including magnesium oxide (MgO) nanoparticles (MgO-NP), could minimize the harmful effects of salinity on plant growth and productivity.

EMs stock solution is an ecofriendly technology and one among the biofertilizers used in this concern (Abd El-Mageed et al., 2020). It contains various fermented mixed cultures of coexisting and mutually compatible microorganisms in an acidic medium, such as the beneficial and nonpathogenic microorganisms (aerobic and anaerobic), including photosynthetic bacteria, lactic acid bacteria, yeast, actinomycetes, and fermenting fungi (Higa and Parr, 1994; Talaat et al., 2015). The stimulating effect of EMs improves soil structure and fertility, organic matter, and nutrient cycling and reduces chemical fertilizers and pesticides dependency, resulting in increased profitability and sustainability (Talaat et al., 2015). Moreover, the combinations of EMs can synthesize bioactive substances such as amino acids, vitamins, sugars, lactic acids, enzymes, and hormones. These compounds promotes plant growth by stimulating root development, photosynthetic capacity, protein activity, germination, flowering, fruiting, and ripening of crops (Higa and Parr, 1994; Hu and Qi, 2013). Additionally, using EMs alleviated the effects of salinity and promoted salt resistance (Talaat, 2015; Abd El-Mageed et al., 2020; Porter et al., 2020). Furthermore, EMs application enhanced nutrient acquisition, accumulation of compatible solutes (soluble sugars, free amino acids, glycine betaine, and proline) (Talaat et al., 2015; Abd El-Mageed et al., 2020), and upregulated the ascorbate–glutathione cycle capacity; a key pathway that reduces oxidative damage (Talaat, 2014). Also, EMs-supplemented soil increased the photosynthetic efficiency of the Photosystem II (PSII), macronutrient contents, and seed yield of bean plants (Iriti et al., 2019).

Magnesium (Mg^2 +^) is a macronutrient involved in several physiological and biochemical processes required for plant growth and development (Jezek et al., 2015). For example, Mg^2 +^ plays an important role in manipulating a crucial biological polyphosphate compound such as ATP, DNA, and RNA (Shinde et al., 2020). Besides, it is the central atom of the chlorophyll molecule and thus essential for activating many enzymes, including ribulose-1,5-bisphosphate-carboxylase/oxygenase (RubisCO). Therefore, Mg^2 +^ plays a crucial role in plant metabolism processes such as sugar synthesis, nutrient uptake, protein biosynthesis, chloroplast formation, phloem loading, and the portioning of photo-assimilates from source to sink organs, and majorly in the light and dark reactions of photosynthesis (Shabala and Hariadi, 2005; Jezek et al., 2015; Feller et al., 2018).

Nanotechnology has recently received much attention as a promising approach in sustainable agricultural applications as an alternative to conventional fertilizers (El-Saadony et al., 2021). At their core, nanoparticles (NPs) have a novel small size (1–100 nm) feature that can alter and assemble atoms that possess and improve physical, chemical, and biological properties. This feature leads to improved performance of the NPs function due to the high surface-to-volume ratio and surface charges (Al-Mamun et al., 2021; El-Saadony et al., 2021). Therefore, nanoscale-basted materials are more reactive than their bulk-scale counterparts, with greater penetration ability into plant tissues and rapid translocation between plant parts (Zahedi et al., 2019; Semida et al., 2021b). Moreover, nanofertilizers ensure the slow and controlled release of the fertilizers and reduce nutrient losses during fertilization, improving the efficient use of nutrients (Rizwan et al., 2019; Al-Mamun et al., 2021). Additionally, available evidence reported that incorporating nanofertilizers of macro- and micronutrients in crop nutrition stimulates the growth and productivity of salt-stressed plants (Mohamed et al., 2017; Adjei et al., 2021; Etesami et al., 2021). Therefore, the development of nanoscale particles of Mg^2 +^ may help to trigger the metabolic pathways, including photosynthesis, leading to better growth and higher yield of plants (Rathore and Tarafdar, 2015).

The coapplication of EMs with nanomaterials such as Mg^2 +^ may be an interesting/novel application to improve sweet potato performance under salinity conditions. Therefore, this study was designed to investigate the coapplication of EMs and MgO-NP on the growth and yield of sweet potato plants cultivated in salt-affected soil. Additionally, this study examines the water status of the tissue, membrane stability, photosynthetic pigments content, nutrients content, accumulation of osmolytes, and antioxidant capacity of sweet potato plants.

## Materials and Methods

### Experimental Site

A number of two field experiments were conducted during the summer of 2020 and 2021 (May to October) at El Fayoum area, Egypt, between latitudes 29°02′ and 29°35′N and longitudes 30°23′ and 31°05′E. The soil was saline sandy loam defined as Typic Torripsamments, siliceous, and hyperthermic (Soil Survey Staff, 1999). Physico-chemical properties of the soil were determined according to the study of Page et al. (1982) and Klute and Dirksen (1986) and are shown in Table 1. The experimental region has a dry summer climate, with average monthly day and night temperatures of 38.2/23.6 and 37.2/22.9°C, respectively, for both seasons. In addition, across both seasons, typical direct solar radiation ranged from 21.8 to 32.8 MJ m^2^ d^1^, and relative humidity ranged from 33.2 to 45.9%.

**TABLE 1:** Some initial chemical properties of the experimental soil (as average for both seasons).

Properties	Unit	Value
Particle size distribution	%	
Sand		76.2
Silt		12.0
Clay		11.8
Texture class		Loamy sand
Bulk density	g cm^–3^	1.55
pH [at a soil: water (w/v) ratio of 1:2.5]		7.65
ECe (at soil – paste extract)	dS m^–1^	7.56
CEC (cation exchange capacity)	cmol_e_ kg^–1^	11.35
CaCO_3_	%	4.80
Organic matter	%	1.10
ESP (exchangeable sodium percentage)		10.66
Available nutrients:		
N	%	0.03
P	mg kg^–1^ soil	5.12
K	mg kg^–1^ soil	55.23
Fe	mg kg^–1^ soil	3.32
Mn	mg kg^–1^ soil	9.00
Zn	mg kg^–1^ soil	0.62
Cu	mg kg^–1^ soil	0.53

### Treatments and Experimental Design

The experimental arrangement was a split-plot system based on randomized complete block design (RCBD) performed in triplicates. The EMs (main plot) were applied with two levels as follows: with (EMs^+^) and without (EMs^–^) in triplicate applications (i.e., at 15, 30, and 45 days after transplantation), and three MgO-NP concentration (0, 50, and 100 μg ml^–1^) were distributed into sub-plots) that were applied foliarly at two times; 30 days after transplantation and 2 weeks later.

In addition to the control (EMs^–^ × MgO NP_0_), there were five treatments as follows: EM^+^ × MgO NP_0_, EMs^–^ × MgO NP_50_, EMs^–^ × MgO NP_100_, EMs^+^ × MgO NP_50_, and EMs^+^ × MgO NP_100_. Supplementary Figure shows the transmission electron microscopy (TEM) image of the produced MgO-NP that were poly-dispersed, with an average size less than 100 nm. Then, 30-day-old vines of sweet potato (*I. batatas* L. cv. Beauregard), obtained from the private farm at Beni Suef governorate, Egypt, were separately transplanted on 2 May 2020 and 10 May 2021, one transplant per emitter, a drip-irrigated system with a one-line and one dripper per plant giving 3.2 L h^–1^. The experimental plot area was 12 m length × 0.70 m row width (8.4 m^2^) and about 0.25 m between plants within row (144 plants per treatment). The cultural, disease, and pest management practices were the same as the local commercial crop production.

### Application of the Effective Microorganisms

Plants were either sprayed with distilled water (EMs^–^) or with EMs (EMs^+^) along with the soil surface at the time of irrigation. The EMs were applied three consecutive times in an EMs formulation that contained a mixture of five groups of beneficial microorganisms: photosynthetic bacteria (*Rhodopseudomonas palustris* and *Rhodobacter sphaeroides*), lactic acid bacteria (*Lactobacillus plantarum*, *Lactobacillus casei*, and *Streptococcus lactis*), yeast (*Saccharomyces cerevisiae* and *Candida utilis*), actinobacteria (*Streptomyces albus* and *Streptomyces griseus*), and fermenting fungi (*Aspergillus oryzae*, *Penicillium* sp., and *Mucor hiemalis*). The mixture was prepared in the Ministry of Agriculture and Land Reclamation (the Centralized Management of Afforestation and the Environment), Giza, Egypt, as an EMs stock solution, which was diluted to 1:1,000 (EM: water, v/v) when used.

### Estimation of the Activity of Enzymatic Antioxidants

Technique, as described by Bradford (1976), was adopted to extract plant tissues as a crude enzyme extract to measure enzymatic and non-enzymatic antioxidant activity. The superoxide dismutase (SOD, EC 1.15.1.1) activity was assessed using the nitro blue tetrazolium (NBT) method of Giannopolitis and Ries (1977), with units defined as the quantity of enzyme required to prevent 50% of the NBT degradation rate at 560 nm. The Aebi (1984) approach was used to determine the catalase (CAT, EC 1.11.1.6) activity, which included a buffer of potassium phosphate (pH 7) and H_2_O_2_ as a substrate. Notably, as H_2_O_2_ is broken down, the absorbance rate at 240 nm decreases, indicating the enzyme activity. According to Rao et al. (1996), the optical density at 290 nm evaluated the ascorbate peroxidase (APX, EC 1.11.1.11) activity. After monitoring the glutathione reductase (GR, EC 1.6.4.1) GSH-dependent oxidation, the cellular activity was assessed as labeled (Rao et al., 1996). After that, three absorbance times were obtained at 340 nm monitoring GSH-dependent oxidation of NADPH.

### Determination of Nonenzymatic Antioxidants

To assess the reduced glutathione (GSH) and ascorbic acid (AsA) concentrations in sweet potato fresh leaves, we used the previously described methods (Griffith, 1980; Mukherjee and Choudhuri, 1983). For determination of GSH, fresh leaf tissue (50 mg) was homogenized in 2 ml of 2% (v/v) metaphosphoric acid, followed by centrifugation at 17,000 × *g* for 10 min. Neutralization of the supernatant (0.9 ml) was performed using 0.6 ml of 10% (w/v) sodium citrate. In each 1.0 ml assay, assessments were performed three times. About 0.3 mM NADPH (700 l), 6 mM 5,5’-dithio-bis-2-nitrobenzoic acid (100 l), distilled water (100 l), and extract (100 l) were used in each assay. After stabilization (at 25°C for 3–4 min), 10 μl of 50 GSH reductase units ml^–1^ was added and the absorbance was then recorded at 412 nm. The AsA was extracted using 10 ml of 6% (w/v) trichloroacetic acid, after which the resulting extract was mixed with 2% (w/v) dinitrophenylhydrazine, thiourea at 10% (w/v) in 70% ethanol (v/v). The mixture was boiled for 15 min and cooled and thereafter was combined with 5 ml of H_2_SO_4_ 80% (v/v). The absorbances were read at 530 nm to quantify the concentration of AsA using a standard curve.

### Osmolytes and Soluble Protein Quantification

The extraction and quantification of free proline (FP) and total soluble sugars (TSS), free amino acids, soluble proteins, and phenolic acid (mg g^–1^ DW) were performed using dry sweet potato leaves. For proline content, assessment was performed according to Bates et al. (1973). Briefly, 0.5 g samples were crushed and grinded using 10 ml sulfosalicylic acid 3% (v/v). The resulting mixtures were centrifuged for 10 min at 10,000 × *g*. About 2 ml of the supernatant was taken into a test tube, and 2 ml of each of ninhydrin solution and glacial acetic acid were placed into the contents of the tube. The tubes were incubated in a water bath at 100°C for 30 min and then transferred to an ice bath to end the reaction. About 5 ml toluene was added to each reaction mixture and vortex-mixed for 15 s. To allow separation of the toluene and aqueous phases, the tubes were left in the dark for at least 20 min at room temperature. The absorbance of each toluene phase was measured at 520 nm after it was carefully collected into a clean test tube. A standard curve made with analytical grade proline was used to evaluate the FP content in each sample. After extraction with 96% (v/v) ethanol, the concentration of TSS in the leaves was measured, as described by Irigoyen et al. (1992). The resultant mixture was heated for 10 min after reacting the extract with an anthrone reagent. A Spectronic Bausch and Lomb 2000 spectrophotometer (Bausch and Lomb analytical systems divisions, Rochester, New York, United States) was used to read the cooled samples at 625 nm. The content of total free amino acids was determined in dry leaves following the method outlined by Rosen (1957). Additionally, soluble proteins and phenolic acid were measured using the techniques recommended by Bradford (1976).

### Physiological Measurements

Chlorophyll *a*, chlorophyll *b*, total chlorophyll, and carotenoid content were extracted and determined (in mg g^–1^ FW; *n* = 9) according to the procedure of Arnon (1949). Fresh leaf samples (0.2 g) were first homogenized in 50 ml 80% (v/v) acetone and then centrifuged at 10,000 × *g* for 10 min. The acetone extract absorbance was measured at 663, 645, and 470 nm using a UV-160 A UV–vis recording spectrometer (Shimadzu, Kyoto, Japan).

The chlorophylls *a* and *b* and total content of carotenoids were calculated as the following equation:


$$\text{Chlorophyll}\,a\,\left(\mathrm{mg}\,{\mathrm{dm}}^{-2}\right)=\left(11.65\times \mathrm{A664}\right)-\left(2.69\times \mathrm{A647}\right)\times \text{v}/\text{sp}$$

$$\text{Chlorophyll}\,b\,\left(\mathrm{mg}\,{\mathrm{dm}}^{-2}\right)=\left(20.8\times \mathrm{A647}\right)-3.14\times \mathrm{A664}\times \text{v}/\text{sp}$$

$$\text{Carotenoids}\,\left(\mathrm{mg}\,{\mathrm{dm}}^{-2}\right)=\left(1,000\,A_{480}-1.28\,\mathrm{chl}\,a-5.67\,\text{chl}\,b\right)\,/\,245\,v/\text{sp}$$

The relative water content (RWC%) was estimated according to Hayat et al. (2007) and calculated (*n* = 9) using the following formula:


$$RWC\left(\%\right)=\left[\frac{\left(F\,M-D\,M\right)}{\left(T\,M-D\,M\right)}\right]\times 100$$

where FM; fresh mass (g), TM; turgid mass (g), DM; dry mass (g). The membrane stability index (MSI)% was measured according to the method of Premachandra et al. (1990). About 0.2 g of total expanded leaves was incubated in water bath at 40°C for 30 min of incubation at 40°, and the solution electrical conductivity was measured (C1). After that, the solution was boiled in water bath at 100°C for 10 min, and then, the MSI was calculated using the following equation.


$$MSI\left(\%\right)=\left[1-\left(\frac{C\,1}{C\,2}\right)\right]\times 100$$

where C_1_, electrical conductivity of the solution at 40°C, and C_2_, the electrical conductivity of the solution at 100°C.

### Measurements of Growth and Yield Characteristics

At the end of the growing season, ten plants were randomly obtained from every experimental plot and assessed for their growth characteristics. First, plant height and stem diameter were recorded, and then, the number of leaves plant^–1^ was counted. Next, the total leaf area plant^–1^ was measured using a digital planimeter, Planix 7 (Sokkia Co., Ltd. Kanagawa, Japan). The plant leaves and branches were then weighed, and their fresh weight was recorded (shoot fresh weight). Also, the shoot dry weight plant^–1^ was recorded after oven-drying at 70°C until constant weight. Finally, five plants of each experimental plot were used to measure the average number of tubers plant^−1^ and total yield hectare^−1^ at the harvest stage.

The water use efficiency (WUE) was calculated as the ratio of fruit yield (kg ha^–1^) and irrigation water applied (m^–3^ ha^–1^) for each irrigation level using the method of Jensen (1983):


$$W\,U\,E=\frac{f\,r\,u\,i\,t\,y\,i\,e\,l\,d\,\left(K\,g\,h\,a^{-1}\right)}{w\,a\,t\,e\,r\,a\,p\,p\,l\,i\,e\,d\,\left(m^{3}\,h\,a^{-1}\right)}$$

### Macronutrient Determination

In the assessment of the macronutrient concentrations in plant tissues (i.e., nitrogen; N, phosphorus; P, and potassium; K^+,^ magnesium; Mg^2 +^, calcium; Ca^2 +^, and sodium; Na^2 +^), sweet potato leaves (*n* = 9) were dried and milled into powder before chemical analysis. Digestion process was performed for the dried leaf samples with a mixture consisting of perchloric and nitric acids (at 1: 3, v/v, respectively). Using a micro-Kjeldahl apparatus (Ningbo Medical Instruments Co., Ningbo, China), N content was determined following the methods of AOAC (2000). The P content was assessed by quantification according to Jackson (1973) using standard reagents of H_2_MoO_7_S, molybdenum blue, diluted H_2_MoO_7_S, and 8% (w/v) NaHSO_3_–H_2_SO_4_. Additionally, the Ca^2+^ and Mg^2+^ contents were assessed using an Atomic Absorption Spectrophotometer Model 3300 (PerkinElmer, Inc., Waltham, MA, United States) as described by Chapman and Pratt (1961). Finally, K^+^ and Na^+^ contents were assessed according to the study of Lachica et al. (1973) in a 50 mg freeze-dried leaf powder suspension and centrifuged at 3,000 × *g* for 10 min at 25°C.

### Statistical Analysis

Microsoft Excel 2016 was used to compute means ± standard error. In addition, the variance analysis for both seasons and error variance homogeneity for all variables was tested. The analysis for the two seasons was performed based on a split-plot in randomized complete block design (RCBD) using GenStat statistical package (version 12) (VSN International Ltd., Oxford, United Kingdom). Means for all variables were separated using Fisher’s least significant difference test at *p* ≤ 0.05 (GENSTAT, 2007).

## Results

### Growth Traits

As shown in Table 2, salinity stress had inhibitory effects on sweet potato growth characteristics, significantly decreasing the shoots fresh weight plant^–1^, shoot dry weight plant^–1^, leaves number, leaf area plant^–1^, and leaves area index. However, salt-stressed sweet potato plants treated with EMs, MgO-NP, and their combinations revealed a considerable improvement in growth traits compared to the untreated stressed plants. In comparison with the control, all single treatments, i.e., EMs^+^, MgO-NP_50_, MgO-NP_100_, increased the growth traits by 22–78%. However, the integrative treatments, EMs^+^ × MgO-NP_50_, EMs^+^ × MgO-NP_100_, caused a higher increase in the growth traits to about 129%. Conclusively, the combined application of EMs^+^ × MgO-NP_100_ resulted in the highest increase in the growth attributes (Table 2).

**TABLE 2:** Effect of foliar application with magnesium oxide (MgO) nanoparticles (MgO-NP) and effective microorganisms (EMs) on growth attributes of sweet potato (*Ipomoea batatas* L.) grown in salt affected soil in 2020 (SI) and 2021 (SII) seasons.

Treatments	Shoots FW plant^–1^ (g)	Shoot DW plant^–1^ (g)	Number of leaves	Leaf area plant^–1^ (dm^2^)	Leaves area index
**SI**					
EM^–^ × MgO NP_0_	687 ± 16.7d	131.3 ± 3.2c	222.5 ± 5.4d	119.9 ± 6.2d	2.99 ± 0.15d
EM^+^ × MgO NP_0_	1183 ± 12.3b	226.3 ± 3.8b	383.4 ± 2.7b	175.3 ± 3.5b	4.38 ± 0.09b
EMs^–^ × MgO NP_50_	1084 ± 13.7c	217.2 ± 2.6b	351.1 ± 4.4c	155.8 ± 2.5c	3.89 ± 0.06c
EMs^–^ × MgO NP_100_	1223 ± 34.5b	233.8 ± 6.6b	396.3 ± 11.2b	183.1 ± 9.8b	4.03 ± 0.24c
EMs^+^ × MgO NP_50_	1517 ± 44.1a	290.0 ± 8.4a	491.4 ± 14.1a	217.9 ± 6.3a	5.45 ± 0.16a
EMs^+^ × MgO NP_100_	1587 ± 33.3a	303.4 ± 6.4a	514.1 ± 10.8a	223.2 ± 5.5a	5.58 ± 0.14a

**SII**					

EM^–^ × MgO NP_0_	737.09 ± 18.3d	142.1 ± 5.3c	230.3 ± 5.4d	126.2 ± 5.1d	3.2 ± 0.16d
EM^+^ × MgO NP_0_	1289 ± 20.1b	233.3 ± 6.9b	369.6 ± 3.7b	163.2 ± 6.3b	4.18 ± 0.10b
EMs^–^ × MgO NP_50_	1177 ± 19.2c	244.2 ± 5.6b	323.2 ± 4.4c	146.3 ± 4.4c	3.66 ± 0.10c
EMs^–^ × MgO NP_100_	1286 ± 32.6b	253.8 ± 7.4b	386.3 ± 11.9b	177.6 ± 5.8b	3.89 ± 0.16c
EMs^+^ × MgO NP_50_	1488 ± 32.1a	310.6 ± 6.4a	473.5 ± 14.3a	200.9 ± 6.8a	5.16 ± 0.21a
EMs^+^ × MgO NP_100_	1590 ± 46.2a	321.4 ± 7.9a	489.2 ± 10.8a	213.2 ± 6.2a	5.23 ± 0.20a

*Values are means ± SE (n = 9). Mean values in each column followed by a different lower-case letter are significantly different by Fisher’s least significant difference test at p ≤ 0.05. FW, fresh weight; DW, dry weight.*

### Yield and WUE

From the results shown in Table 3, cultivating sweet potato in salt-affected soil induced negative impacts on yield components and recorded the lowest values. However, EMs and/or MgO-NP application greatly attenuated the salt-induced adverse effects of sweet potato plants’ yield and components. Contextually, all single treatments, EMs^+^, MgO-NP_50_, and MgO-NP_100_, increased the tubers number plant^−1^ (by 38, 30, and 40%), tuber weight (by 41, 34, and 38%), tuber weight plant^−1^ (by 75, 56, and 77%), and tuber yield (by 44, 25, and 38%), respectively, relative to the untreated plants. Interestingly, coapplication of EMs^+^ and MgO-NP proved higher effectiveness since the EMs^+^ × MgO-NP_50_ and EMs^+^ × MgO-NP_100_ treatments elevated the abovementioned yield traits by 67 and 76%, 47 and 49%, 109 and 122%, and 70 and 80%, respectively, compared to the untreated salt-stressed plants. This increase in tuber yield was significant in the WUE rise by 71 and 82% with the EMs^+^ × MgO-NP_50_ and EMs^+^ × MgO-NP_100_ treatments, respectively (Table 3).

**TABLE 3:** Effect of foliar application with magnesium oxide (MgO) nanoparticles (MgO-NP) and effective microorganisms (EMs) on yield and yield components and water use efficiency (WUE) of sweet potato (*Ipomoea batatas* L.) grown in salt affected soil in 2020 (SI) and 2021 (SII) seasons.

Treatments	Number of tubers plant^–1^	Tuber weight (g)	Tubers weight plant^–1^ (g)	Tuber yield (t ha^–1^)	WUE (Kg m^–3^)
**SI**					
EM^–^ × MgO NP_0_	5.57 ± 0.36d	98.9 ± 4.8b	559 ± 19.4e	16.97 ± 2.48e	2.08 ± 0.10d
EM^+^ × MgO NP_0_	7.67 ± 0.33bc	134.9 ± 6.5a	1030 ± 17.3c	25.75 ± 1.43c	3.14 ± 0.08b
EMs^–^ × MgO NP_50_	7.00 ± 0.29c	130.2 ± 3.6a	912 ± 16.1d	22.79 ± 2.15d	2.78 ± 0.05c
EMs^–^ × MgO NP_100_	7.67 ± 0.33bc	134.1 ± 6.7a	1024 ± 16.3c	24.59 ± 1.16c	2.99 ± 0.04c
EMs^+^ × MgO NP_50_	9.00 ± 0.53ab	137.9 ± 7.8a	1233 ± 13.7b	30.82 ± 1.34b	3.76 ± 0.12a
EMs^+^ × MgO NP_100_	9.77 ± 0.58a	135.6 ± 6.1a	1307 ± 25.4a	32.67 ± 1.36a	3.98 ± 0.13a

**SII**					

EM^–^ × MgO NP_0_	5.22 ± 0.44d	100.0 ± 4.2c	603 ± 20.2e	18.07 ± 2.62e	2.28 ± 0.05e
EM^+^ × MgO NP_0_	7.23 ± 0.43bc	144.9 ± 4.6b	1002 ± 16.3c	24.75 ± 2.37c	3.09 ± 0.06c
EMs^–^ × MgO NP_50_	6.99 ± 0.36c	136.2 ± 4.2b	900 ± 14.1d	20.80 ± 2.99d	2.74 ± 0.02d
EMs^–^ × MgO NP_100_	7.45 ± 0.45bc	141.1 ± 5.3b	1033 ± 15.2c	23.69 ± 2.44c	3.07 ± 0.02c
EMs^+^ × MgO NP_50_	8.99 ± 0.39ab	153.9 ± 6.6ba	1188 ± 20.3b	28.66 ± 2.634b	3.69 ± 0.04b
EMs^+^ × MgO NP_100_	9.20 ± 0.66a	160.6 ± 5.1a	1269 ± 26.2a	30.1 ± 3.03a	3.92 ± 0.02a

*Values are means ± SE (n = 9). Mean values in each column followed by a different lower-case letter are significantly different by Fisher’s least significant difference test at p ≤ 0.05.*

### Tissue Water Status, Membrane Integrity, and Photosynthetic Pigments

Sweet potato plants grown under salinity stress showed a significant decrease in the leaf water status (RWC), cell membrane integrity (MSI), and photosynthetic pigments (chlorophylls *a* and *b*, total chlorophylls, and carotenoids) contents (Table 4). Nevertheless, exogenously applied EMs and/or MgO-NP lessened salt-induced damage to the tissue water status, membrane integrity, and photosynthetic pigments (Table 4). The integrative EMs^+^ × MgO-NP_50_ and EMs^+^ × MgO-NP_100_ revealed the highest ameliorative effects. They significantly increased the RWC by 17 and 19%, MSI by 14 and 15%, chlorophyll *a* by 66 and 81%, chlorophyll *b* by 143 and 168%, total chlorophylls by 108 and 131%, and carotenoids by 168 and 198%, respectively, compared with the salt-stressed plants without elicitors (Table 4).

**TABLE 4:** Effect of foliar application with magnesium oxide (MgO) nanoparticles (MgO-NP) and effective microorganisms (EMs) on the concentrations of leaf photosynthetic pigments and plant water status of sweet potato (*Ipomoea batatas* L.) grown in salt affected soil in 2020 (SI) and 2021 (SII) seasons.

	RWC	MSI	Chl *a*	Chl *b*	Total Chl	Car
				
Treatments	%		(mg mL^–1^)			
**SI**						
EM^–^ × MgO NP_0_	70.48 ± 0.51d	65.81 ± 0.69d	0.97 ± 0.03d	1.19 ± 0.16d	2.16 ± 0.16d	0.12 ± 0.01c
EM^+^ × MgO NP_0_	79.51 ± 0.94bc	71.56 ± 0.65bc	1.31 ± 0.18c	2.31 ± 0.29c	3.62 ± 0.47c	0.20 ± 0.06b
EMs^–^ × MgO NP_50_	77.98 ± 1.4c	71.52 ± 0.51bc	1.23 ± 0.03c	2.23 ± 0.11c	3.45 ± 0.17c	0.24 ± 0.03b
EMs^–^ × MgO NP_100_	79.41 ± 0.74bc	70.44 ± 0.22c	1.39 ± 0.05bc	2.52 ± 0.12b	3.92 ± 0.17bc	0.25 ± 0.01b
EMs^+^ × MgO NP_50_	82.63 ± 0.88ab	73.77 ± 0.51a	1.61 ± 0.12ab	2.91 ± 0.16a	4.52 ± 0.27ab	0.36 ± 0.01a
EMs^+^ × MgO NP_100_	84.31 ± 1.2a	74.14 ± 0.57a	1.76 ± 0.13a	3.11 ± 0.28a	5.00 ± 0.38a	0.38 ± 0.01a

**SII**						

EM^–^ × MgO NP_0_	71.63 ± 0.50d	64.44 ± 1.3d	1.00 ± 0.09e	1.20 ± 0.09d	2.20 ± 0.17d	0.14 ± 0.02e
EM^+^ × MgO NP_0_	80.88 ± 1.2b	70.11 ± 0.98bc	1.36 ± 0.01c	2.13 ± 0.23c	3.49 ± 0.39c	0.19 ± 0.01d
EMs^–^ × MgO NP_50_	77.32 ± 1.6c	71.63 ± 0.68bc	1.20 ± 0.12d	2.34 ± 0.24c	3.54 ± 0.37c	0.24 ± 0.01c
EMs^–^ × MgO NP_100_	80.31 ± 0.99b	70.99 ± 1.1c	1.41 ± 0.21b	2.69 ± 0.18b	4.10 ± 0.34bc	0.26 ± 0.03c
EMs^+^ × MgO NP_50_	83.96 ± 1.3a	74.23 ± 0.67a	1.66 ± 0.16ab	2.89 ± 0.19b	4.55 ± 0.43ab	0.33 ± 0.06b
EMs^+^ × MgO NP_100_	84.63 ± 2.3a	74.99 ± 0.93a	1.80 ± 0.19a	3.29 ± 0.23a	5.09 ± 0.49a	0.39 ± 0.08a

*Values are means ± SE (n = 9). Mean values in each column followed by a different lower-case letter are significantly different by Fisher’s least significant difference test at p ≤ 0.05. RWC, relative water content; MSI, membrane stability index; Chl a, chlorophyll a; Chl b, chlorophyll b; total Chl, total chlorophyll; car, carotenoids.*

### Nutrient Acquisition

In both growing seasons, P, N, K^+^, Ca^2+^, and Mg^2+^ contents were significantly decreased in the untreated sweet potato plants when exposed to soil with salinity conditions (Table 5). In contrast, the salt-stressed plants treated with EMs and/or MgO-NP showed increased P, N, K^+^, Ca^2+^, and Mg^2+^ content concentration. Additionally, the integrative treatments were most effective, exceeding all individual treatments. Particularly, the P, N, K^+^, Ca^2+^, and Mg^2+^ contents increased by 57 and 63%, 72 and 80%, 44 and 47%, 55 and 105%, 138 and 193% corresponding to the coapplication of EMs^+^ × MgO-NP_50_ and EMs^+^ × MgO-NP_100_, respectively, when compared to the control treatment (Table 5). Finally, a significant increase in Na^+^ concentration in the leaves and a decline in the K^+^/Na^+^ ratio of the untreated plants were observed due to salinity stress response (Table 5). The application of EMs and/or MgO-NP alleviated the adverse effects of salt stress, decreased the leaves content from Na^+^, and accordingly reduced the K^+^/Na^+^ ratio (Table 5).

**TABLE 5:** Effect of foliar application with magnesium oxide (MgO) nanoparticles (MgO-NP) and effective microorganisms (EMs) on the concentrations of leaf photosynthetic pigments and plant water status of sweet potato (*Ipomoea batatas* L.) grown in salt affected soil in 2020 (SI) and 2021 (SII) seasons.

Treatments	P	N	K^+^	Ca^2 +^	Na^+^	Mg^2 +^	K^+^/Na^+^ ratio
**(mg g DW** ^–^ **^1^)**							
**SI**							
EM^–^ × MgO NP_0_	19.88 ± 1.66e	2.89 ± 0.10e	20.76 ± 0.43d	6.33 ± 0.33c	12.60 ± 0.54a	2.16 ± 0.17e	1.65 ± 0.01e
EM^+^ × MgO NP_0_	24.23 ± 1.04c	3.66 ± 0.11c	29.57 ± 0.21ab	11.67 ± 0.33ab	8.89 ± 0.21e	3.17 ± 0.15d	3.13 ± 0.10a
EMs^–^ × MgO NP_50_	21.66 ± 2.3d	3.22 ± 0.12d	28.41 ± 0.64c	10.83 ± 1.2ab	9.85 ± 0.10d	4.83 ± 0.20c	2.83 ± 0.05b
EMs^–^ × MgO NP_100_	23.09 ± 1.37c	3.55 ± 0.13c	29.36 ± 0.13ab	9.83 ± 0.83b	9.69 ± 0.10d	5.43 ± 0.16b	3.10 ± 0.01a
EMs^+^ × MgO NP_50_	28.23 ± 1.84b	5.02 ± 0.12a	29.14 ± 0.30b	8.33 ± 0.17bc	7.8 ± 0.11b	5.53 ± 0.18b	3.74 ± 0.01d
EMs^+^ × MgO NP_100_	31.27 ± 1.18a	4.99 ± 0.13a	29.91 ± 0.52a	14.00 ± 2.0a	7.35 ± 0.11c	6.32 ± 0.23a	4.07 ± 0.02c

**SII**							

EM^–^ × MgO NP_0_	18.69 ± 1.66d	2.57 ± 0.09e	19.22 ± 0.13d	6.33 ± 0.33b	14.35 ± 0.20a	1.99 ± 0.13e	1.34 ± 0.01c
EM^+^ × MgO NP_0_	23.96 ± 1.04b	3.38 ± 0.15c	27.86 ± 0.34ab	12.00 ± 1.0a	9.64 ± 0.32c	2.86 ± 0.12d	3.07 ± 0.10a
EMs^–^ × MgO NP_50_	20.03 ± 2.3c	3.02 ± 0.16d	25.36 ± 0.43c	9.83 ± 0.83a	11.41 ± 0.32b	3.03 ± 0.18c	2.49 ± 0.01b
EMs^–^ × MgO NP_100_	24.30 ± 1.37b	3.23 ± 0.09c	27.26 ± 0.47ab	10.67 ± 0.33a	10.33 ± 0.54bc	4.63 ± 0.22b	2.85 ± 0.01ab
EMs^+^ × MgO NP_50_	32.11 ± 1.84a	4.36 ± 0.14b	28.22 ± 0.26b	11.33 ± 1.7a	9.85 ± 0.54bc	4.36 ± 0.29b	2.97 ± 0.13a
EMs^+^ × MgO NP_100_	31.55 ± 1.18a	4.81 ± 0.16a	28.98 ± 0.13a	11.67 ± 0.67a	10.60 ± 0.80bc	5.88 ± 0.36a	2.86 ± 0.22ab

*Values are means ± SE (n = 9). Mean values in each column followed by a different lower-case letter are significantly different by Fisher’s least significant difference test at p ≤ 0.05. Dw, dry weight.*

### Osmolytes Content

**FIGURE 1:**
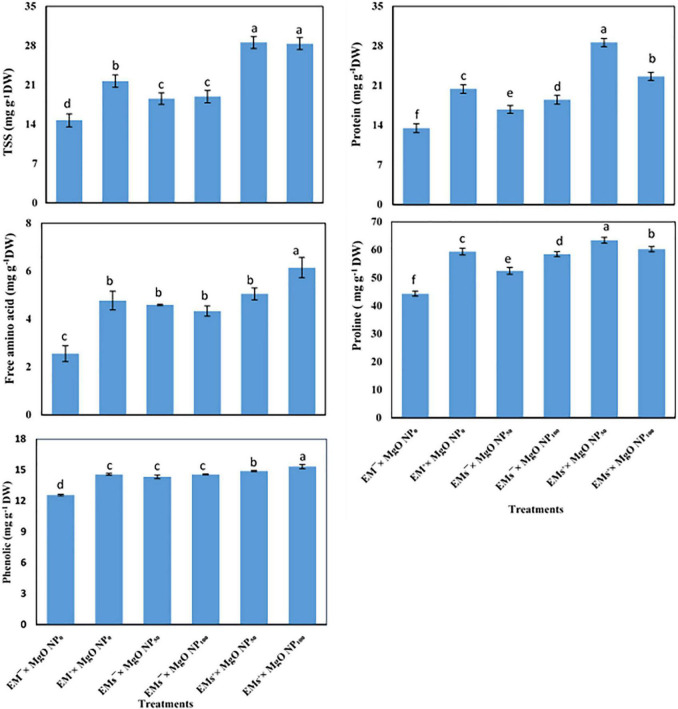
Interactive effect of effective microorganisms (EMs) interacted with foliar application with magnesium oxide (MgO) nanoparticles (MgO-NP) on total soluble sugars (TSS), proteins, free amino acids, proline, and phenolics of sweet potato (*Ipomoea batatas* L.) as average for both seasons. Different letters on the bars refer to significant differences among means based on Fisher’s least significant difference test at the *p* < 0.05 level.

TSS, total free amino acid, and proline concentration increased under salt stress and further increased when sweet potato plants were exposed to the exogenous application of EMs and/or MgO-NP (Figure 1). This observation showed that coapplication of EMs^+^ × MgO-NP_50_ and EMs^+^ × MgO-NP_100_ treatments recorded the highest values. The untreated control markedly enhanced the TSS by 94 and 93%, total free amino acid by 98 and 141%, and proline by 43 and 36%, respectively. Salt stress significantly decreased the sweet potato protein and phenolic content and mediated the protein and phenolic concentration improvement whereas externally applied EMs and/or MgO-NP were adopted (Figure 1). The single treatments increased protein and phenolic by 25–51 and 6–7%. However, the integrative treatments, EMs^+^ × MgO-NP_50_ and EMs^+^ × MgO-NP_100_, significantly increased these characteristics by 10 and 13%, and 112 and 68%, respectively, compared with the control (Figure 1).

### Antioxidants: Enzymatic Activities and Non-enzymatic Contents

**FIGURE 2:**
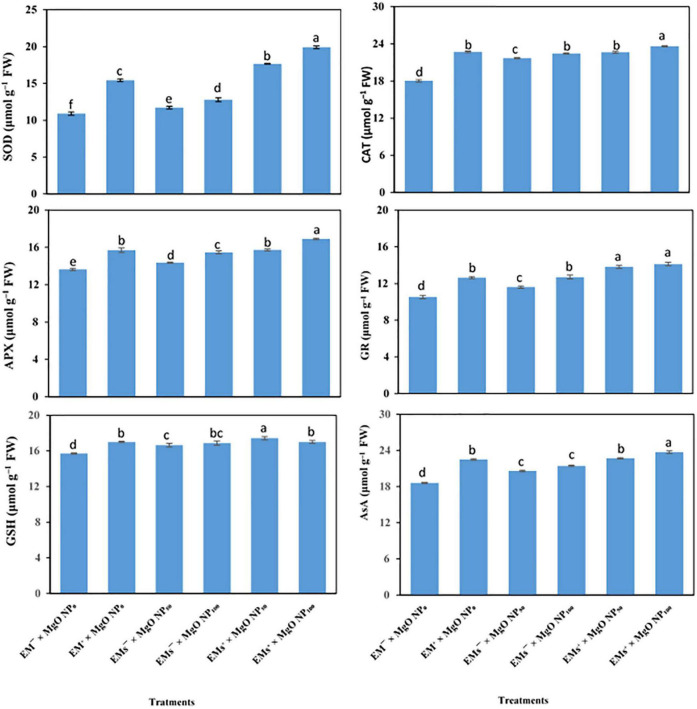
Interactive effect of effective microorganisms (EMs) interacted with foliar application with magnesium oxide (MgO) nanoparticles (MgO-NP) on enzymatic [e.g., superoxide dismutase (SOD), catalase (CAT), ascorbate peroxidase (APX), glutathione reductase (GR), glutathione (GSH) and ascorbate (AsA)] antioxidants activity of sweet potato (*Ipomoea batatas* L.) as average for both seasons. Different letters on the bars refer to significant differences among means based on Fisher’s least significant difference test at the *p* < 0.05 level.

Sweet potato plants grown under salinity stress exhibited lower antioxidant capacity (both non-enzymatic; AsA and GSH, and enzymatic; SOD, CAT, APX, and GR antioxidant, Figure 2). Nevertheless, the application of EMs and/or MgO-NP to salt-stressed sweet potato plants upregulated the activity of enzymatic (SOD, CAT, APX, and GR) and increased the concentration of non-enzymatic (GSH and AsA) antioxidants (Figure 2). However, all single treatments (*i.e.*, EMs^+^, MgO-NP_50_, and MgO-NP_100_) improved the analyzed antioxidative compounds. Additionally, the coapplication of EMs^+^ × MgO-NP_50_ and EMs^+^ × MgO-NP_100_ was more effective in alleviating salt-induced damages to *I. batatas*. Compared to the stressed control plants, EMs^+^ × MgO-NP_50_ and EMs^+^ × MgO-NP_100_ treatments increased the AsA by 22 and 27%, GSH by 11 and 8%, SOD by 62 and 83%, APX by 15 and 24%, GR by 31 and 34%, and CAT by 26 and 31%, respectively (Figure 2).

## Discussion

In this study *I. batatas* plants were cultivated in salt-affected soil (7.56 dS m^–1^; Table 1), causing several morphological and physio-biochemical function abnormalities. Sweet potato plants responded to the salt stress by reducing growth traits (Table 2), tuber yield (Table 3), tissue water status (RWC), index of membrane integrity (MSI), photosynthetic pigments (Table 4), and acquisition of nutrients (Table 5). This retardation of plant growth and productivity results from salinity, lowering the soil water potential, which reduces the water uptake by the roots (Sharma and Garg, 2021). Consequently, this impaired cell division, expansion, metabolism, ion imbalance, stomatal closure, gas exchange, and reduced tissue water content, including increased oxidative stress indicators over their scavenging antioxidants and deteriorated photosynthesis-linked pigments (Gupta and Huang, 2014; Arif et al., 2020; Rady et al., 2021b). Plants have developed various stress-responsive mechanisms to withstand salt stress, such as activating the antioxidative compounds, including enzymatic and non-enzymatic antioxidants functioning in tandem with osmolytes (Ahanger et al., 2017; Semida et al., 2020). However, the plant’s endogenous defense system is insufficient to relieve salinity stress under severe stress conditions, necessitating the addition of exogenous stimuli to help salt-stressed plants to adapt (Rady et al., 2021a).

The application of EMs or/and MgO-NP in the current study showed effective mechanisms for reducing salinity stress’s harmful effects. Also, due to their properties, the coapplication of EMs and MgO-NP may boost the physio-biochemical pathways against salt stress. In salt-stressed sweet potato plants, exogenously-applied EMs or/and MgO-NP (50 or 100 μg ml^–1^) improved the morpho-physiological, biochemical, and productivity responses compared to the control plants. EMs or/and MgO-NP application also alleviated the adverse effects of salinity stress on the growth parameters of sweet potato plants, thereby promoting tuber yield and WUE compared to stressed control plants. The coapplication of EMs and MgO-NP assigned the highest positive effects, achieving the best results. These restorations in plant growth and productivity of sweet potato by application of EMs or/and MgO-NP are probably due to the improvements in growth-linked molecules of osmolytes and antioxidants that help plants recover from salt stress (Talaat, 2015). Furthermore, EMs could promote plant performance by synthesizing bioactive substances, including vitamins, amino acids, sugars, lactic acid, hormones, and enzymes (Higa and Parr, 1994; Hu and Qi, 2013).

The MSI detects cell membrane stability whereas RWC estimates the plant water status in terms of the physiologically available water in plant tissues (Slabbert and Krüger, 2014; Abd El-Mageed et al., 2019; Abdelkhalik et al., 2019); however, both were reduced under salt stress in the current study (Table 4). Thus, maintaining higher RWC in stress tissues maintains cell physiological functions (i.e., photosynthesis, stomatal aperture, gas exchange, and cell expansion and development), which proceed *via* osmoregulation as an effective stress tolerance mechanism (Abid et al., 2018; Desoky et al., 2021). Coapplication of EMs and MgO-NP improved the recovery of tissues in salt-stressed sweet potato by increasing the tissue water content (RWC; Table 4). This response may be due to the effect of EMs and MgO-NP in elevating the accumulation of osmolytes (e.g., soluble sugars, FP, amino acids, and K^+^ ion; Figure 1 and Table 5) and the antioxidative compounds (Figure 1). These modulated the osmotic pressure and mitigated the oxidative damage, preserving cellular turgor pressure and membrane integrity against stress damage (Talaat, 2014; Talaat et al., 2015; Cai et al., 2018).

Exogenous applied EMs and/or MgO-NP enhanced salt tolerance in stressed sweet potato plants, since enhanced the membrane integrity (Table 4). Higher enzymatic antioxidant activities, as well as GSH and AsA levels, as a result of EM and/or MgO-NP treatments (Figure 1), are linked to reduced ROS-induced oxidative damage and minimize lipid peroxidation, which help in cell membrane stabilization and function maintenance (Gomathi and Rakkiyapan, 2011). Under salinity stress, the buildup of Ca^2+^ in plant tissues by applying EMs and MgO-NP (Table 5) inhibits Na^+^ uptake and transport, as well as binding to the cell wall and increased cell membrane stability (Rahman et al., 2016).

Salinity stress decreased the cellular physiological functions, including photosynthetic pigment levels, due to the restriction in chlorophyll biosynthesis that elevates chlorophyll degradation (by chlorophyllase enzyme) or/and insufficient nutrient uptake (Sarker and Oba, 2020; Muhammad et al., 2021). Salinity stress could also be linked to oxidative damage to the chlorophyll pigments caused by ROS in the chloroplast and interferes with Na^+^ and Cl^–^ ions with the protein pigment complexes (Sarker and Oba, 2020). Photosynthesis is an important physio-chemical process in plants, with its efficiency mainly related to the changes in the number of photosynthetic pigments under salinity conditions (Arif et al., 2020). Therefore, increasing the chlorophylls and carotenoids content is employed as a biochemical signal of salt stress tolerance (Stefanov et al., 2016). Our results illustrated that the combined supplementation of EMs and MgO-NP significantly enhanced the chlorophylls (*a* and *b*) and carotenoid levels in salt-stressed sweet potato plants (Table 4). Magnesium is at the core of all chlorophylls involved in the activation/activity of Rubisco and, therefore, an essential component of photosynthesis (Moynier and Fujii, 2017; Hauer-Jákli and Tränkner, 2019). Its presence may be the sole cause of the stimulated response in the MgO-NP-treated plants. EMs were observed to relieve salinity-induced damages to the photosynthetic efficiency of *Vicia faba* and *Phaseolus vulgaris* plants by modifying several physiological processes such as maintaining cell turgor, membrane integrity, nutrient acquisition, levels of osmolytes, and antioxidant capacity (Talaat et al., 2015; Iriti et al., 2019). Besides acting as auxiliary light-harvesting pigments, carotenoids act as an antioxidant that protects the photosynthesis system by aiding the heat dissipation of excess excitation energy in the photosynthetic machinery, thus preventing superoxide generation (Hashimoto et al., 2016; Abid et al., 2018). Therefore, increasing the chlorophylls (*a* and *b*) and carotenoids in treated plants indicates the stimulatory role of EMs and MgO-NP under salinity stress.

This current study showed that soil salinity induced ionic imbalance in the cells of sweet potato plants. It reduced P, N, K^+^, Mg^2+^, and Ca^2+^ acquisition, but increased Na^+^ accumulation (Table 5) attributed to changes in ionic homeostasis, potential nutrients uptake, and specific toxic ion (Parida and Das, 2005; Talaat et al., 2015). Besides their importance in plant growth and productivity, K^+^ acts as an osmoregulator in plants under abiotic stress (Assaha et al., 2017). However, the increased Na^+^ concentration in plant tissues competitively affected the K^+^ uptake since both share similar transport channels (Farooq et al., 2018; Isayenkov and Maathuis, 2019), thus reducing the cytosolic K^+^/Na^+^ ratio (Table 5). This resultant effect also provokes the disruption of cellular homeostasis, oxidative stress, nutrient deficiency, interference among K^+^ and Ca^2+^ functions, and hampered growth (Assaha et al., 2017). Interestingly, coapplication of EMs and MgO-NP mediated recovery of ionic homeostasis and nutrient uptake, decreasing Na^+^ level, whereas increasing P, N, K^+^, and Ca^2+^ acquisition in salt-stressed sweet potato. A crucial salinity tolerance mechanism is maintaining a balanced cytosolic K^+^/Na^+^ ratio (Farooq et al., 2018) achieved by the integrative application of EMs and MgO-NP. Further, increased Ca^2+^ and Mg^2+^ levels in the supplemented plants with EMs and MgO-NP would assist stressed plants in preventing Na^+^ accumulation in the cell to a lethal level, as indicated by Talaat et al. (2015).

Ion analysis demonstrated the amelioration of the nutrient status in Mg-treated plants in terms of Mg^2 +^, K^+^, and Mn in different *Zea mays* organs, minimizing the adverse effects of salt stress (Jezek et al., 2015). Our results conform to Kanjana (2020), who observed positive effects by exogenous application of Mg-NP in increasing the nutrient (N, P, K^+^, and Mg^2 +^) concentration in cotton plants. However, the application of EMs exhibited a favorable effect on nutrient acquisition in salt-stressed sweet potato plants. The application of EMs probably provokes such a response due to their involvement in promoting tissue water content and cell membrane integrity (Table 4). Furthermore, EMs supplementation stimulated root growth of *Phaseolus vulgaris* plants under salinity stress that enhanced the potential nutrient uptake (Talaat et al., 2015). Finally, EMs promote the decomposition of organic materials and improve the mineralization of organic matter, releasing more nutrients into the soil for plant absorption (Hu and Qi, 2013).

Salinity composes stress by damaging ionic and osmotic balances in plants. Our study shows that the plant’s defense machinery, including accumulation of osmolytes (Figure 1) and upregulating the activity of antioxidant molecules (Figure 2), was enhanced by the integrative effects of EMs and MgO-NP, which enabled the stressed plants to withstand the salinity stress. Similarly, osmotic adaptation is vital for maintaining cell turgor, essential for growth and productivity. Therefore, plants synthesize several osmolytes under salt stress, such as soluble sugars, total free amino acids, and FP. The coapplication of EMs and MgO-NP significantly increased the free amino acids, TSS, and FP levels of osmotically-stressed sweet potato plants compared to the control plants (Figure 1). These osmolytes may assist salt stress tolerance in sweet potato plants by promoting osmotic adjustment, therefore stimulating root water uptake by aiding its diffusion into the cells, thereby maintaining cellular turgor, and increasing RWC (Table 4), consequently allowing the plant to continue the physiological processes under salinity stress (Abid et al., 2018; Desoky et al., 2021). Moreover, the osmolytes effectively scavenge the ROS and stabilize the protein and membrane integrity (Arif et al., 2020), consequently increasing MSI (Table 5). Our results also showed that salt stress-induced physiological disorders in sweet potato plants reduced the phenolic and protein concentration. This result correlates with the reports of Talaat (2015) that salt stress reduced total protein levels by increasing protein hydrolysis and lowering protein synthesis enzymes activity. However, our results demonstrated that the application of EMs and MgO-NP ameliorated salt-induced inhibitory effect on protein and phenolic levels, given that protein and phenolic content increased in EMs and MgO-NP-treated plants compared to the control. This enhancement in protein level may be linked to increased nutrient acquisition and higher K^+^ accumulation by applying EMs and MgO-NP under salt stress, which helps to maintain a higher ratio of K^+^/Na^+^, thus preventing the inhibitory effect of salt stress in various enzymes, including protein biosynthesis (Talaat, 2015). Similarly, phenolic molecules have antioxidant properties for scavenging ROS, including a significant reported link between phenolic compounds and abiotic-stress tolerance as an effective predictor of the level of redox state maintenance in salinity-stressed cells (Kiani et al., 2021; Šamec et al., 2021). Therefore, it is worth noting that increasing protein and phenolic content can be effective ways for EMs and MgO-NP-treated plants to achieve salinity tolerance.

Under salinity stress, ROS overproduction induces oxidative damage, therefore activating the antioxidant machinery, including enzymatic and non-enzymatic, in the plants (Sarker and Oba, 2020). Our results revealed that coapplication of EMs and MgO-NP upregulated the activity of AsA, GSH, SOD, APX, GR, and CAT (Figure 2). These antioxidative compounds (enzymatic and non-enzymatic), soluble sugars, proline, phenolic compounds, and photosynthetic auxiliary pigments such as carotenoids, are the powerful ROS quenchers and scavengers, making them effective salinity mitigators (Zhang and Dai, 2019). Additionally, externally applied EMs mediated an increased activity of the enzymatic antioxidants and the AsA and GSH in bean plants, indicating an improved ascorbate–glutathione cycle (AsA-GSH) as a potent mechanism in the detoxification of oxidative stress indicators; H_2_O_2_ and MDA (Talaat, 2014). Furthermore, MgO-NP application boosted tobacco plant growth and increased the activity of SOD and peroxidase (POD) enzymes (Cai et al., 2018). Accordingly, our findings demonstrated the importance of applying EMs and/or MgO-NP to relieve the salinity stress on the sweet potato.

## Conclusion

The results of present study clearly showed that the application of EMs and/or MgO-NP alleviates the inhibitory effects of salt stress on sweet potatoes. It also showed the coapplication of EMs and MgO-NP as a more effective method exceeding all individual approaches. Furthermore, the integrative application of EMs and MgO-NP enhanced the antioxidant activity (AsA, GSH, SOD, APX, GR, and CAT) and osmolytes accumulation (FP, total free amino acids, soluble sugars, K^+^) as well as increased the phenolic and protein contents. Additionally, the coapplication of EMs and MgO-NP induced improvements in RWC, MSI, photosynthetic pigments (chlorophylls *a* and *b*, and carotenoids), and nutrient acquisition, consequently promoting the growth and productivity of salt-stressed sweet potato. Moreover, the results suggested that biostimulants, i.e., EMs and MgO-NP, should be used in future applications to improve plant performance (growth and productivity) under salinity conditions.

## Data Availability Statement

The original contributions presented in the study are included in the article/Supplementary Material, further inquiries can be directed to the corresponding authors.

## Author Contributions

TAA, MG, KH, SAQ, KE-T, and AA conceived and designed the research. TAA, SAQ, and KE-T supervised the study. TAA, MG, KH, ME-S, SAE-M, and AA performed field experiments. TAA, MG, KH, ME-S, SAE-M, and AA developed the biochemical and physiological analyses. TAA, SAQ, and KE-T analyzed the data. TAA, MG, KH, SAE-M, HA, and AA assisted with experiments and/or data evaluation. All authors contributed to the article and approved the submitted version.

## Conflict of Interest

The authors declare that the research was conducted in the absence of any commercial or financial relationships that could be construed as a potential conflict of interest.

## Publisher’s Note

All claims expressed in this article are solely those of the authors and do not necessarily represent those of their affiliated organizations, or those of the publisher, the editors and the reviewers. Any product that may be evaluated in this article, or claim that may be made by its manufacturer, is not guaranteed or endorsed by the publisher.
