# Supplementary material for: Coapplication of Effective Microorganisms and Nanomagnesium Boosts the Agronomic, Physio-Biochemical, Osmolytes, and Antioxidants Defenses Against Salt Stress in Ipomoea batatas
Source: Front Plant Sci. 2022 Jul 13;13:883274. doi: 10.3389/fpls.2022.883274 (PMC9326395; doi:10.3389/fpls.2022.883274)
Supplement: Supplementary file 1 [file Data_Sheet_1.pdf]

Supplementary material

Coapplication of Effective Microorganisms and Nanomagnesium Boosts the Agronomic, Physio-biochemical, Osmolytes, and Antioxidants Defenses against Salt Stress in *Ipomoea batatas*

Taia A. Abd El-Mageed, Mohammed A.H. Gyushi, Khaulood A. Hemida, Mohamed T. El-Saadony, Shimaa A. Abd El-Mageed, Synan F. AbuQamar[*], Khaled A. El-Tarabily[*] and Abdelsattar Abdelkhalik

*** Correspondence:**
Synan AbuQamar: sabuqamar@uaeu.ac.ae
Khaled El-Tarabily: ktarabily@uaeu.ac.ae

Supplementary material

Supplementary Figure S1. Transmission electron microscopy image of magnesium oxide (MgO) nanoparticles (MgO-NP).

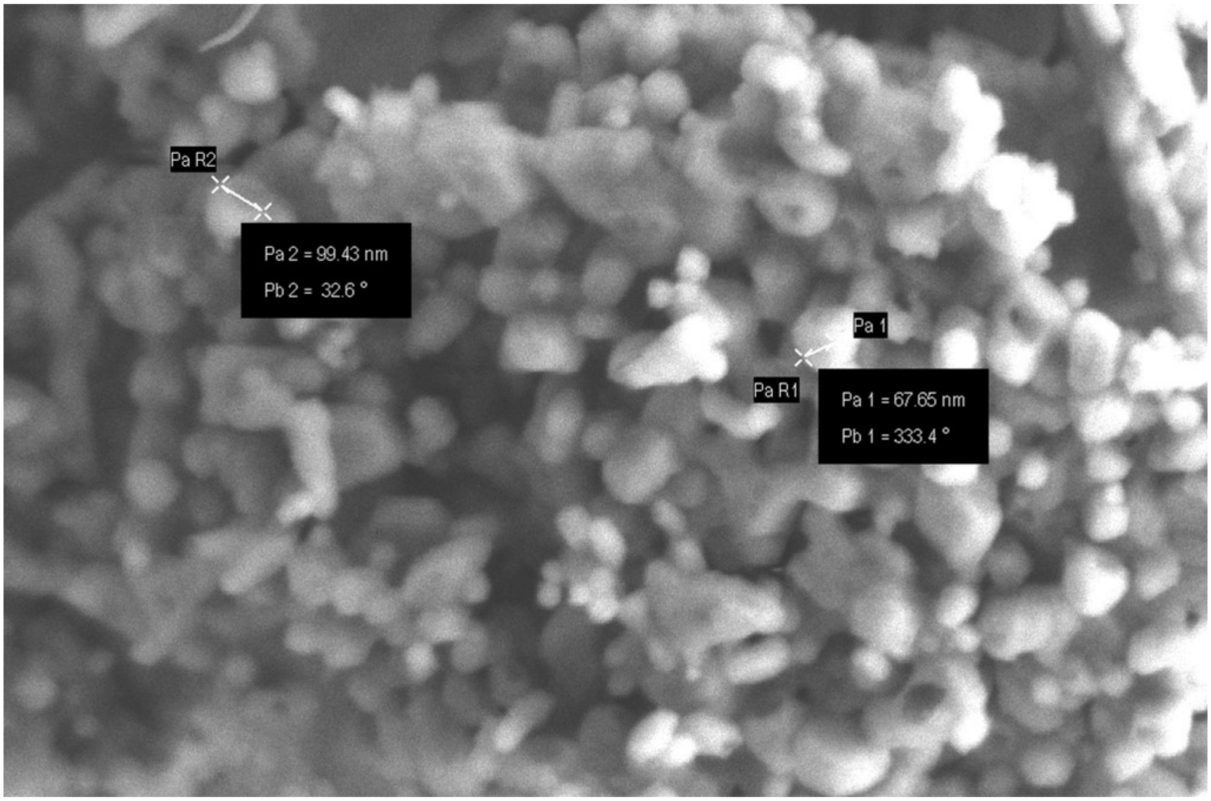

Supplementary Figure S1. Transmission electron microscopy image of magnesium oxide (MgO) nanoparticles (MgO-NP).
